# The level of claudin-7 is reduced as an early event in colorectal carcinogenesis

**DOI:** 10.1186/1471-2407-11-65

**Published:** 2011-02-10

**Authors:** Jette Bornholdt, Stine Friis, Sine Godiksen, Steen S Poulsen, Eric Santoni-Rugiu, Hanne C Bisgaard, Inger MB Lothe, Tone Ikdahl, Kjell M Tveit, Egil Johnson, Elin H Kure, Lotte K Vogel

**Affiliations:** 1 Department of Cellular and Molecular Medicine, Faculty of Health Science, University of Copenhagen, Denmark; 2 Department of Biology, Faculty of Science, University of Copenhagen, Copenhagen, Denmark; 3 Department of Medical Anatomy, Faculty of Health Science, University of Copenhagen, Copenhagen, Denmark; 4 Department of Pathology, Diagnostic Center, Copenhagen University Hospital, Copenhagen, Denmark; 5 Department of Pathology, Oslo University Hospital, Oslo, Norway; 6 Department of Oncology, Oslo University Hospital, Oslo, Norway; 7 Department of Gastroenterological Surgery Oslo University Hospital, Oslo, Norway; 8 Faculty of Medicine, University of Oslo, Oslo, Norway; 9 Department of Genetics, Institute for Cancer Research, Oslo University Hospital, Oslo, Norway; 10 Department of Environmental and Health Studies, Telemark University College, Bø, Norway

## Abstract

**Background:**

Compromised epithelial barriers are found in dysplastic tissue of the gastrointestinal tract. Claudins are transmembrane proteins important for tight junctions. Claudins regulate the paracellular transport and are crucial for maintaining a functional epithelial barrier. Down-regulation of the oncogenic serine protease, matriptase, induces leakiness in epithelial barriers both *in vivo *and *in vitro*. We found in an *in-silico *search tight co-regulation between *matriptase *and *claudin-7 *expression. We have previously shown that the *matriptase *expression level decreases during colorectal carcinogenesis. In the present study we investigated whether *claudin-7 *expression is likewise decreased during colorectal carcinogenesis, thereby causing or contributing to the compromised epithelial leakiness of dysplastic tissue.

**Methods:**

The mRNA level of *claudin-7 *(CLDN7) was determined in samples from 18 healthy individuals, 100 individuals with dysplasia and 121 colorectal cancer patients using quantitative real time RT-PCR. In addition, immunohistochemical stainings were performed on colorectal adenomas and carcinomas, to confirm the mRNA findings.

**Results:**

A 2.7-fold reduction in the *claudin-7 *mRNA level was found when comparing the biopsies from healthy individuals with the biopsies of carcinomas (p < 0.001). Reductions in the *claudin-7 *mRNA levels were also detected in mild/moderate dysplasia (p < 0.001), severe dysplasia (p < 0.01) and carcinomas (p < 0.01), compared to a control sample from the same individual. The decrease at mRNA level was confirmed at the protein level by immunohistochemical stainings.

**Conclusions:**

Our results show that the *claudin-7 *mRNA level is decreased already as an early event in colorectal carcinogenesis, probably contributing to the compromised epithelial barrier in adenomas.

## Background

DNA damage promotes carcinogenesis. This is clearly seen when DNA repair mechanisms are compromised. The eukaryotic cell takes many measures to prevent DNA damage, including formation of physical barriers to prevent the entry of carcinogens and other substances into the organism. In the gastrointestinal tract, a layer of polarised epithelial cells, held together by tight junctions and covered by a layer of mucus, forms a surprisingly efficient barrier. However, this barrier is often compromised already in dysplastic tissue [1]. This is likely to be a factor driving carcinogenesis by allowing carcinogens to enter the underlying tissue.

In the intestine, molecules may pass the monolayer of epithelial cells, either by the transcellular route involving transcytosis or by the paracellular route crossing the tight junctions. Tight junctions are primarily located at the apical end of the lateral plasma membrane [2]. In addition to controlling the paracellular diffusion, tight junctions prevent the diffusion of membrane proteins and lipids between the apical and the basolateral plasma membrane domains [3]. Tight junctions are formed mainly by three types of integral proteins: occludins, junctional adhesion molecules and claudins [4]. In total, 24 different mammalian claudins have been described [5]. The composition of claudins in the tight junction complex is thought to dictate the permeability of the epithelium by regulating the permeability of the tight junctions [4,6]. The expression levels of various claudins in a tissue can change according to physiological and pathological conditions, thereby altering the permeability of the epithelial barrier [7].

Connections of tight junctions between cells are made by interactions between the extracellular loops of the claudins. However, the molecular mechanisms and the exact stoichiometry behind the assembly of tight junctions are poorly understood [8]. The majority of claudins are known to increase the epithelial tightness by sealing the tight junctions. Claudins like claudin-1, -4, -5, -7, -8, -11, -14, and -19 are considered sealing claudins, as increased expression of these leads to increased epithelial tightness [6,9-12]. Other claudins are able to form paracellular anion- and cation pores as well as water channels [5,13]. Claudin-2, -10, and -16 are examples of pore-forming claudins known to decrease epithelial tightness when expression is increased.

The serine protease matriptase plays an important but, as yet, poorly defined role in the generation and maintenance of epithelial barriers. Matriptase, encoded by the *ST14 *gene, is expressed in most epithelial cells [14] and its proteolytic activity is tightly regulated by at least two membrane-bound inhibitors, HAI-1 (*SPINT1*) and HAI-2 (*SPINT2*) [15-17]. Ablation of the *ST14 *gene in mice generates a phenotype with compromised epithelial barrier function and fatal outcome [18,19]. In addition, dysregulated matriptase expression as investigated in transgenic mice has been shown to have a very strong oncogenic potential [15]. A study using the colonic adenocarcinoma cell line Caco-2, which spontaneously form tight monolayers of polarized cells, when grown on filters, showed that siRNA-induced down-regulation of matriptase resulted in compromised epithelial barrier function [20].

We have previously shown that the mRNA level of *matriptase *and its inhibitors are significantly down-regulated during colorectal carcinogenesis [21,22]. Still, the molecular mechanism whereby the proteolytic activity of matriptase affects the epithelial barrier is unknown. In the search for downstream effectors of matriptase, we made an *in-silico *array based study to identify genes co-regulated with the matriptase gene, *ST14*. The analysis showed that besides the already known inhibitor of matriptase, HAI-1 encoded by *SPINT1, CLDN7*, encoding *claudin-7*, is the gene most tightly co-regulated with the *ST14 *gene. Claudin-7 belongs to the class of claudins promoting epithelial tightness [10-12] and is found in most epithelia, for instance in the airways [23], the intestines [24,25], the Loop of Henle and the collecting duct of the kidney [8]. Claudin-7 is involved in regulation of the permeability of Cl^- ^and Na^+ ^ions. Recently, claudin-7 knockout mice were generated and shown to have a normal phenotype at birth. However, within days they developed chronic dehydration, leading to a fatal outcome 12 days after birth [12].

In the present study, we investigated the levels of *claudin-7 *mRNA during colorectal carcinogenesis and found that *claudin-7 *mRNA is significantly decreased in mild/moderate dysplasia, severe dysplasia and carcinomatous tissue. The decrease in *claudin-7 *level could also be confirmed at the protein level.

## Methods

### *In-silico *co-expression array database study

The COXPRESSdb version 4.0 located at http://coxpresdb.jp/[26] was used to identify genes co-regulated with matriptase. All expression data for this database are based on affymetrix GeneChip, information which has been released by NCBI GEO. The analysis was performed using default settings entering *ST14 *as gene symbol in the gene search tool. In the results, the *ST14 *icon under the human gene search was selected. Expression similarity for *ST14 *in relation to all other genes in the database was calculated using Pearson's correlation coefficient and ranked. The opposite correlation coefficients where also calculated and ranked. To evaluate the strength of co-expression a mutual ranking (MR) value was calculated using the formula MR_(AB) _= (rank_(A→B)_* rank_(B→A))_^0.5^. The lower the MR value the higher the co-expression.

### Human tissue samples

The tissues used in this study have previously been described [22]. In short, the KAM cohort is based on a screening performed in the Norwegian Colorectal Cancer Prevention study (NORCCAP) in the county of Telemark, Norway [27] with the ID number NCT00119912 at Clinicaltrials.gov. In addition, a series of colorectal cancer (CRC) cases was recruited to the cohort from Telemark Hospital in Skien and Ulleval University Hospital in Oslo. The KAM study is approved by the Regional Committee for Medical Research Ethics and the Norwegian Data Inspectorate. In the present study, we analysed 121 cases with carcinoma, 100 cases with adenomas and 18 healthy individuals. From individuals with adenomas, control tissue was sampled 30 cm above the anus. From patients with carcinomas, two control samples were taken from the surgically removed specimen. One sample was taken adjacent to the cancer (normal adjacent) and the other sample was taken as distant from the cancer as possible (normal distant). The histology of the adenomas was examined independently by two histopathologists, who categorised the degree of dysplasia as either mild/moderate (n = 87) or severe (n = 13). Consensus was reached in all cases. Cases of dysplasia were also classified as either low- or high-risk according to the size and/or differentiation state of the adenoma. A high-risk adenoma is defined as an adenoma measuring ≥ 10 mm in diameter and/or with villous components or showing severe dysplasia [27]. Carcinomas were classified according to Dukes staging. The distribution of gender and age among individuals included in the study is shown in Table 1.

**Table 1:** Characteristics of cases and healthy individuals participating in this study

	Healthy individuals	Individuals with mild/moderate dysplasia	Individuals with severe dysplasia	Individuals with carcinomas
Total number of individuals in the study	(n = 18)	(n = 87)	(n = 13 )	(n = 121)
Number of men	6	61	7	66
Number of women	12	26	6	55

Mean age ± S.D.	56.8 ± 4.5	56.9 ± 3.6	54.9 ± 3.1	69.5 ± 12.0

Comparing the group of healthy individuals with the other groups showed that there were significantly more males in the group with mild dysplasia and CRC (p < 0.001; Chi Squared test). The mean age is significantly higher among patients with CRC compared to the three other groups (p < 0.001; Kruskal-Wallis and Dunn's Multiple Comparison test).

### Real-time reverse transcriptase polymerase chain reaction

The tissue samples were frozen as soon as possible after surgery and stored in liquid nitrogen until RNA purification. Total RNA was purified from tissue as recommended by the manufacturer using E.Z.N.A. Total RNA Kit II (cat. no. R6834-02, Omega Bio-Tek) and the RNase Free DNase kit I (cat. no. E1091-01). cDNA synthesis was performed on approximately 200 ng RNA per 20 μl using the High-Capacity cDNA Archive Kit (cat. no. 4375222, Applied Biosystems). Quantitative real time RT-PCR for *claudin-7 *was performed on the ABI7300 sequence detection system (Applied Biosystems) in Universal PCR Master Mix (cat. no. 4326614, Applied Biosystems) using 250 nM probe and 300 nM primers. Primers and probe were: *CLDN7 *forward 5'-ATGATGAGCTGCAAAATGTACGA-3'; *CLDN7 *reverse 5'-GCACCAGGGAGACCACCAT-3'; *CLDN7 *probe 5'- FAM-CGCCCTGTCCGCGGCCTT-BHQ-3'. Primers were designed within different exons and with the probe covering the border of exon 1 and exon 2 to prevent amplification of genomic DNA. Primers and probes were obtained from TAGCopenhagen (Denmark). The endogenous *β-actin *control was obtained pre-developed (cat. no.4310881E) from Applied Biosystems. In a validation experiment using a control sample, a dilution series was assayed by the comparative C_t _method [28]. When C_t _values were plotted against the logarithmic value of the amount of cDNA added, it was shown that the assays were quantitative over a range of 4096-fold dilution and that the PCR reactions had similar efficiencies provided that a threshold of 0.1 is used for *claudin-7 *and 0.175 is used for *β-actin*. The threshold is a fixed fluorescence signal level above the baseline and the C_t _value of a sample is determined as the fractional cycle number where the sample's fluorescence signal exceeds the threshold. All samples were quantified in triplicates. The standard deviation on the triplicates was 6% or less. The standard deviation on repeated measurements of the same sample (the control) in separate experiments was 26%, indicating the day-to-day variation of the assay. Negative controls (where the RNA was not converted into cDNA) and positive controls were included in all runs. Samples for which either the *β-actin *or *claudin-7 *values fell outside the upper or lower limits of the standard curve were excluded from the study.

### Western blot analysis

25-50 mg of frozen tissue was lysed in 500 μl PBS containing 1% Triton X-100, 0.5% deoxycholate, and protease inhibitors (10 mg/l benzamidine, 2 mg/l pepstatin A, 2 mg/l leupeptin, 2 mg/l antipain, and 2 mg/l chymostatin). Protein concentrations were measured using BCA™ Protein Assay Kit (cat. no. 23225, Pierce). Samples were mixed with 2 × SDS PAGE sample buffer containing DTT and boiled for 5 min. The proteins were resolved on 7% acrylamide gels and transferred to Immobilon-P PVDF membranes (Millipore cat. no. IPVH00010). The blots were blocked with 10% non-fat dry milk in PBS containing 0.1% Tween-20 (PBST). PVDF membranes were probed with 0.5 μg/ml rabbit anti-claudin-7 antibody (cat. no. 34-9100, Invitrogen) in 1% non-fat dry milk in PBST at 4°C overnight. Membranes were washed 3 × 5 min wash with PBST and incubated 1 hour with 2 ng/ml goat anti-rabbit secondary antibody conjugated with horseradish peroxidase (HRP) (cat no. 1858415, Pierce) diluted in 1% non-fat dry milk in PBST. After 3 × 5 min wash with PBST the signal was developed using the ECL reagent Supersignal West Femto Maximum Sensitivity Substrate (cat. no. 34095, Pierce) according to the protocol supplied by the manufacturer, and developed with a Fuji LAS1000-camera (FujiFilm, Sweden). Blots were stripped in 5 ml Restore™ Western Blot Stripping Buffer (cat. no. 21059, Pierce) for 15 min at 37°C. The above mentioned procedure was repeated, using 0.3 μg/ml primary antibody against ß-actin (cat. no. 8226, Abcam) for one hour at room temperature and 2 ng/ml goat anti-mouse secondary antibody conjugated with HRP (cat. no. 1858413, Pierce) for one hour.

### Immunohistochemistry

Paraformaldehyde-fixed, paraffin-embedded tissue sections were used for the immunohistochemical analysis. The sections were deparaffinated and antigen retrieval was performed in 10 mM Tris, 0.5 mM EGTA, and pH 9 by microwave treatment for 18 min at 600 Watt. Subsequently, after cooling for 20 min they were rinsed with distilled water. The sections were then treated with 3% H_2_O_2 _in distilled water for 10 min and rinsed thoroughly in distilled water, washed in TBS buffer pH 7.6 for 5 min before a 10 min incubation with 1% BSA diluted in TBS buffer. Subsequently, the sections were incubated at room temperature in rabbit anti-claudin-7 antibody (cat. no. 34-9100, Invitrogen) 2 μg/ml in 1% BSA in TBS buffer for 60 min. The sections were then washed 3 × 5 min in TBS buffer, before visualization in EnVision™+ System HRP/rabbit (cat. no. K-4011, DAKO) for 30 min. After an additional 3 × 5 min wash in TBS buffer, DAB (cat. no. K-4011, DAKO) was applied for 10 min. The slides were then rinsed in distilled water and counterstained with Mayer's Haematoxylin for 90 sec and rinsed in water for 5 min before dehydration and mounting in Pertex™ Mounting Medium (cat. no. 00801, Histolab).

### Statistical analysis

GraphPad Prism 4 was used for the statistic calculations. The data were not adjusted for gender since the incidence ratio of CRC between the genders is 1:1 in Norway. For all statistical analysis the data was log transformed. Kruskal Wallis and Dunn's Multiple Comparison test were used for statistical comparison of samples from healthy individuals, samples of mild/moderate dysplasias, severe dysplasias and CRC samples. Paired Student's t-test was used for comparison of affected tissue with the matching control sample.

## Results

### *In-silico *analysis of genes co-expressed with the matriptase gene, *ST14*

**Figure 1:**
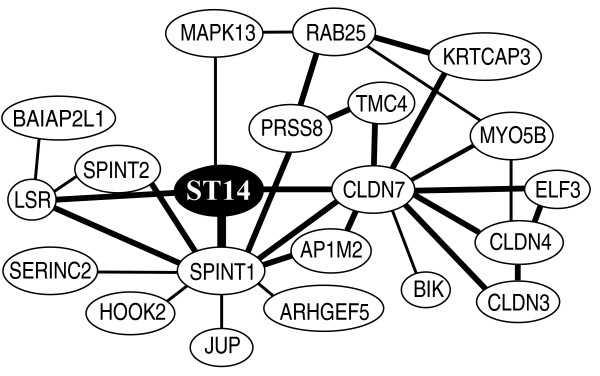
***In-silico *analysis showing the ST14 co-expressed gene network**. The figure shows a modified version of the analysis performed using the COXPRESdb version 4.0 http://coxpresdb.jp/. Each gene is represented by a node. Lines between nodes indicate co-expression. The genes with direct ST14 co-expression are marked with a line to the ST14 node. These genes are *SPINT1 *(encoding HAI-1), *CLDN7 *(encoding claudin-7), *LSR *(encoding lipolysis stimulated lipoprotein receptor) and *MAPK13 *(encoding mitogen-activated protein kinase 13). Bold lines indicate a mutual ratio (MR) below 5 and normal lines indicate a MR between 5 and 30.

In order to identify genes co-regulated with the ST14 gene, encoding matriptase, an *in-silico *analysis was performed. The analysis, performed on COXPRESSdb version 4.0 database, shows a network of co-expressed genes including the *ST14 *gene (Figure 1). Many of these genes are already known to be either inhibitors or substrates of matriptase, such as *SPINT1 *and *SPINT2*, encoding the inhibitors of matriptase, HAI-1 and HAI-2 respectively, and the *PRSS8 *gene encoding the matriptase substrate, prostasin. *ST14 *has the highest degree of co-regulation with its inhibitor *SPINT1 *(correlation coefficient = 0.70 (Pearson's) and a MR = 2.0). In addition to these previously described molecules in the matriptase pathway, we found a new gene, *CLDN7*, that encodes claudin-7 (correlation coefficient = 0.69 and MR = 2.8) to be co-regulated with the ST14 gene. Moreover, the analysis showed that *claudin-7 *and *matriptase *have conserved co-expression between human and mouse orthologs. As claudin-7 is involved in the regulation of the epithelial permeability, we found it plausible that the increased epithelial leakiness observed during carcinogenesis could be mediated through a decreased level of claudin-7.

### Expression of *claudin-7 *mRNA during carcinogenesis

**Figure 2:**
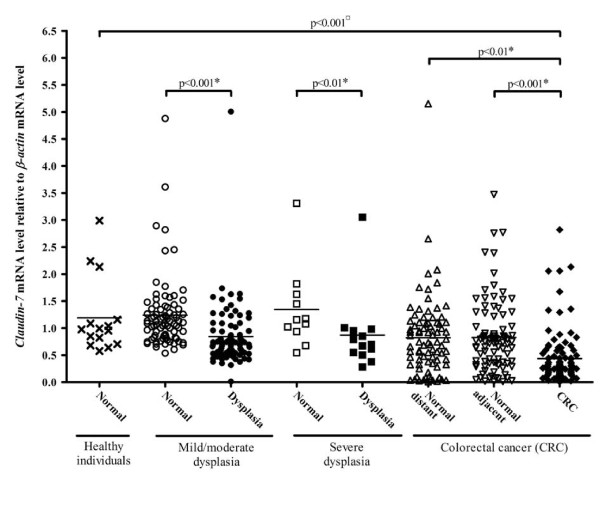
***Claudin-7 *mRNA levels in healthy individuals, individuals with dysplasia and individuals with carcinomas as determined by real-time RT-PCR**. Samples from healthy individuals (cross), normal (open circle) and affected tissue (filled circle) from individuals with mild/moderate dysplasia, normal (open square) and affected tissue (filled square) from individuals with severe dysplasia, normal adjacent (open triangle), normal distant (open triangle) and carcinomatous tissue (filled diamond) from colorectal cancer patients were analysed for *claudin-7 *mRNA levels relative to the *β-actin *mRNA levels. The horizontal line represents the mean values. The p-value indicated with a ¤ is calculated using Kruskal Wallis and Dunn's Multiple Comparison test and p-values marked with a * are calculated using Paired Student's t-test.

The mRNA levels of *claudin-7 *were measured by quantitative real-time RT-PCR. We successfully measured *claudin-7 *mRNA in 15 biopsies from normal mucosa in healthy individuals, in 84 mild/moderate dysplasias with 82 corresponding control samples, 13 severe dysplasias with 11 corresponding control samples, and in 102 colonic carcinomas with 82 corresponding distant and 93 corresponding adjacent control samples (Figure 2 and Table 2).

**Table 2:** *Claudin-7 *mRNA levels in normal and affected tissues normalised to the *β-actin *mRNA level.

Variable	mRNA level in normal tissue Mean ± S.D.	**P**^**a**^	mRNA level in adenomas/carcinomas Mean ± S.D.	**P**^**a**^	**P**^**b**^
Healthy individuals	1.19 ± 0.70				

Individuals with mild/moderate dysplasia	1.24 ± 0.67	NS	0.84 ± 0.58	NS	<0.001

Individuals with severe dysplasia	1.34 ± 0.75	NS	0.87 ± 0.69	NS	<0.01

Cancer patients	0.82 ± 0.73 (distant) 0.83 ± 0.66 (adjacent)	NS NS	0.44 ± 0.48 "	<0.001	<0.01 <0.001

NS = not significant.

a) p value for the comparison to the expression levels in tissue from healthy individuals using Kruskal Wallis and Dunn's Multiple Comparison test.

b) p value for the comparison of the expression levels in normal and affected tissue from the same individual using Paired Student's t-test.

In general, our analysis showed that the *claudin-7 *mRNA levels were decreased as an early event in the carcinogenesis, as the *claudin-7 *mRNA level is decreased already in the mild/moderate dysplasias. The decrease was maintained in the severe dysplasias and the carcinomas. The levels of *claudin-7 *mRNA in normal mucosa from individuals with dysplasia or carcinomas were not affected (Table 2). A highly significant 2.7-fold reduction in the *claudin-7 *mRNA level was found, when comparing the biopsies from healthy individuals with the samples of carcinomas (p < 0.001, Kruskal Wallis and Dunn's Multiple Comparison test). Comparing affected and normal tissue from the CRC patients using Paired Student's t-test showed a 2.0-fold reduction (95% confidence interval (CI): 1.4-2.9) and a 1.7-fold reduction (CI: 1.2-2.5) in expression level for normal adjacent (p < 0.001) and normal distant (p < 0.01) samples, respectively. A similar analysis on individuals with mild/moderate or severe dysplasia showed a 1.6-fold (CI: 1.4-1.9; p < 0.001) and a 1.5-fold (CI: 1.2-1.9; p < 0.01) reduction in the *claudin-7 *mRNA levels, respectively. We found no correlation between the *claudin-7 *mRNA levels and age, gender, high risk/low risk status of the adenoma, or Duke's stage of the carcinoma (data not shown).

### Expression and localisation of claudin-7 protein in colorectal cancers

**Figure 3:**
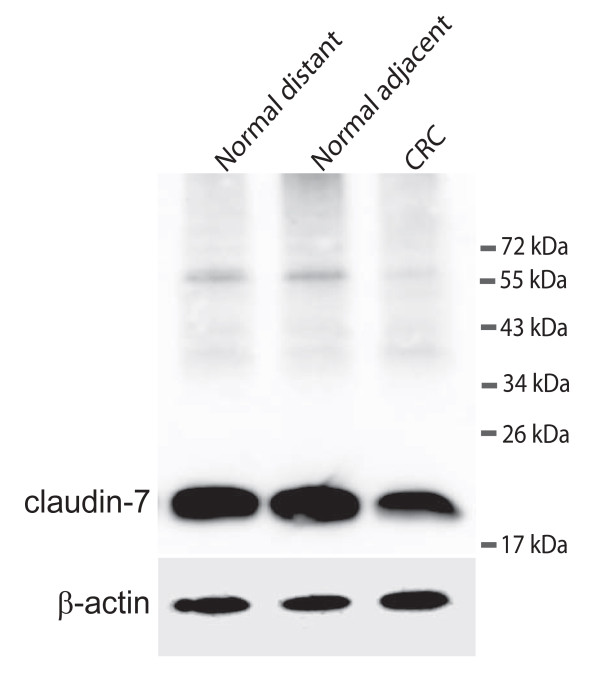
**Western blot detecting claudin-7 and ß-actin**. The upper panel shows claudin-7 in samples from a colorectal cancer patient. Normal distant is a sample taken as far away from the tumour as possible in the surgically removed tissue. Normal adjacent is a sample taken just adjacent to the tumour and CRC is from the cancer itself. The lower panel shows the loading control (ß-actin).

**Figure 4:**
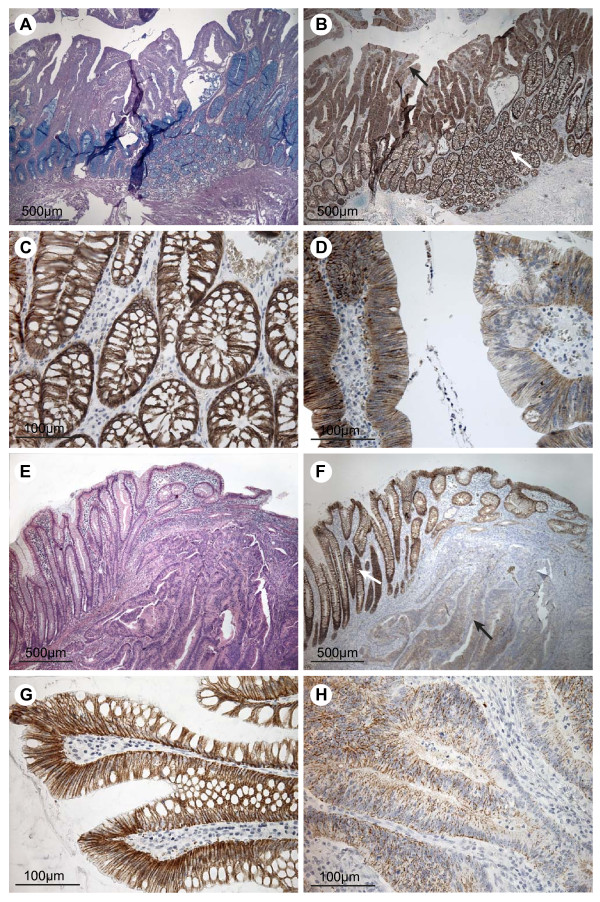
**Immunohistochemical staining for claudin-7 in colorectal adenomas, carcinomas and adjacent normal tissue**. The top four pictures are taken from an individual with dysplasia but no record of carcinoma and the bottom four pictures are from a patient with colorectal cancer. A) Tissue section including both normal mucosa and dysplastic tissue, stained with PAS/Alcian. B) Neighbouring tissue section stained for claudin-7. The white arrow indicates mucosa of normal appearance and the black arrow indicates dysplastic tissue. C) High magnification of the normal mucosa from B, showing staining mainly at the basolateral cell membranes of the epithelial cells. D) High magnification of the dysplastic tissue from B showing a patchy staining pattern with areas of low and normal staining. E) Tissue section with cancerous tissue to the right and normal mucosa to the left, stained with Hematoxylin and Eosin. F) Neighbouring tissue section stained for claudin-7. The white arrow indicates the mucosa with normal appearance. The black arrow points out carcinomatous tissue. G) High magnification of histologically normally appearing mucosa from F, showing staining mainly localised to the basolateral cell membranes of the surface epithelial cells. H) High magnification of carcinomatous tissue showing faint, patchy staining of the epithelial strands of tumour tissue. Scale bars: 500 μm (A + B + E + F), 100 μm (C + D + G + H).

Carcinoma tissue and normal mucosa from five CRC patients were extracted and the proteins were analysed on Western blots (Figure 3). Only one distinct band of approximately 22 kDa was detected using an antibody against claudin-7. This is in accordance with the expected size of claudin-7 [29]. Immunohistochemical stainings were performed on adenomas from four individuals without any history of carcinomas and on CRC tissue and normal mucosa from five CRC patients (Figure 4 and additional files 1). All stainings of adenomas showed essentially the same, and a representative example is shown in Figure 4. An intense claudin-7 reaction was seen in histologically normal appearing mucosa (Figure 4B and 4C) whereas dysplastic tissue ranged from almost no staining to staining similar to the normal mucosa (Figure 4B and 4D). Stainings of the five colorectal cancer samples also showed essentially the same and is shown in figure 4 and additional file 2. Histologically normal mucosa from cancer patients also displayed an intense claudin-7 reaction that was detected from the transitional zone (Figure 4F and 4G). In the cancerous tissue, the staining was reduced or almost absent (Figure 4F and 4H). These observations are consistent with the *claudin-7 *mRNA levels obtained in this study. The normal appearing mucosa from the transitional zone showed, at high magnification, staining for claudin-7. The staining was mainly localised at the basolateral plasma membranes of the surface epithelial cells (Figure 4C and 4G), whereas the ademomatous and carcinomatous tissue showed faint, patchy staining of the epithelial strands of tumor tissue (Figure 4B and 4F).

## Discussion

In the present study we found a correlation between the *claudin-7 *mRNA level, as determined by real-time RT-PCR, and the claudin-7 protein level, as determined by immunohistochemistry in normal mucosa, adenomas and carcinomas of the colon. This suggests that *claudin-7 *mRNA level reflects the protein level. Our results suggest that a decrease in the level of *claudin-7 *occurs already in mild/moderate dysplasias as an early event in carcinogenesis and that the decreased level is maintained in severe dysplasias and in the CRC tissue. To our knowledge this is the first analysis of *claudin-7 *mRNA expression in colorectal mucosa from healthy individuals and from individuals with colorectal dysplasia. Previous studies have compared normal and cancerous colorectal tissue from the same individual. The group of Nakayama et al. [24] found a lower expression of claudin-7 in 80% of invasive CRCs (n = 90) than in non-neoplastic tissue, which corresponds well with our findings. However, our observations are in contrast to the studies by Kuhn et al. [25] and Darido et al. [30], who both found higher expression of claudin-7 protein in CRC tissue as compared to normal tissue from the same individual, using immunohistochemistry alone.

For other types of cancer, a number of studies have compared claudin-7 expression in malignant tissue and normal tissue from patients. They find that claudin-7 is down-regulated in head and neck cancer [31], nasopharyngeal cancer [32], squamous cell carcinomas of the oesophagus [33] and in breast cancer [29,34,35]. However, claudin-7 seems to be up-regulated in gastric cancer [36] and ovarian cancer [37]. In squamous cell carcinomas of the oesophagus, reduced expression of claudin-7 correlates with invasion and metastasis [33]. Likewise, reduced claudin-7 levels correlates with the histological grading of breast carcinomas [29,34].

Our *in-silico *analysis suggests that dysregulated matriptase may affect the epithelial tightness during carcinogenesis by modulating the expression of claudin-7. We have previously shown that the mRNA expression levels of *matriptase (ST14) *and *HAI-1 *(*SPINT1*) are dysregulated during colorectal carcinogenesis in a cohort very similar to the one used in this study [21,22]. Comparison between the mRNA levels of *claudin-7 *(this study) and *matriptase *mRNA levels in the similar cohort [21] shows that they have a virtually identical pattern, confirming that mRNA expression of *matriptase *and *claudin-7 *are closely correlated.

This suggests that the genes encoding *matriptase *and *claudin-7 *may be regulated by the same transcription factors. Alternatively, the expression levels of matriptase may affect the expression levels of claudin-7. We attempted to analyse whether an over-expression of matriptase in the colonic adenocarcinoma cell line Caco-2 influenced the *claudin-7 *mRNA level. However, these experiments were inconclusive as manipulation of matriptase expression is cytotoxic (Vogel et al., unpublished results). Further investigations are needed to clarify this point.

It has recently been shown that siRNA silencing of matriptase in Caco-2 cells resulted in up-regulation of the claudin-2 protein level [20]. Claudin-2 is a pore-forming claudin closely related to claudin-7 [38]. Up-regulation of claudin-2 thus results in increased epithelial leakiness. Claudin-2 is heavily up-regulated in colorectal cancer tissue compared to normal tissue from the same individual [39,40]. There have been no reports about the expression level of claudin-2 in dysplastic tissue. The increased epithelial permeability seen in colorectal dysplastic tissue is thus probably the result of dysregulation of a number of claudins, some of which may depend on matriptase expression or activity.

In esophageal squamous cell carcinoma cells, over-expression of claudin-7 resulted in more adhesive and less invasive cells, whereas knockdown of *claudin-7 *using a small interfering RNA approach led to enhanced invasion into a three-dimensional matrix [41]. This suggests that *claudin-7 *down-regulation does indeed contribute to drive carcinogenesis.

## Conclusions

In conclusion, the present study shows that the level of claudin-7 is decreased as an early event in colorectal carcinogenesis and may play a role by decreasing epithelial tightness, thereby allowing carcinogens to enter the tissue.

## Competing interests

The authors declare that they have no competing interests.

## Authors' contributions

SF and LV conceived the idea of the study. SF performed the *in-silico *analysis. EHK, IMBL, EJ, TI and KMT established the NORCCAP and Ulleval colorectal cancer cohort (KAM cohort). LV and EHK extracted the RNA. LV validated primers and probes. JB did the western blotting. ESR performed the immunohistochemical stainings. SSP took the pictures. IMBL performed and evaluated the sections of immunohistochemical analysis. JB analysed the data, prepared the figures and performed the statistical analysis. JB drafted the first manuscript. All authors helped with the draft, read and approved the final version.

## Pre-publication history

The pre-publication history for this paper can be accessed here:

http://www.biomedcentral.com/1471-2407/11/65/prepub

## Supplementary Material

Additional file 1 **Immunohistochemical staining for claudin-7 in dysplastic tissue**. The figure shows sections from three individuals with dysplasias. Each individual is represented in the rows marked with the numbers id#62, id#488 and id#536. The left column shows an area in the biopsy with mucosa of normal histological appearance, whereas the right column shows an area of dysplasia. Scalebars: 100 μm. Click here for file

Additional file 2 **Immunohistochemical staining for claudin-7 in colorectal cancer**. The figure represents colorectal cancer sections from four patients. Each individual is represented in the rows marked with the numbers patient A, patient C, patient D and patient O. The left column shows an area in the biopsy with mucosa of normal histological appearance, whereas the right column shows an area of cancerous tissue. Scalebars: 100 μm Click here for file
