# Supplementary figures and images for: The level of claudin-7 is reduced as an early event in colorectal carcinogenesis
Source: BMC Cancer. 2011 Feb 10;11:65. doi: 10.1186/1471-2407-11-65 (PMC3045986; doi:10.1186/1471-2407-11-65)

## Slide 1
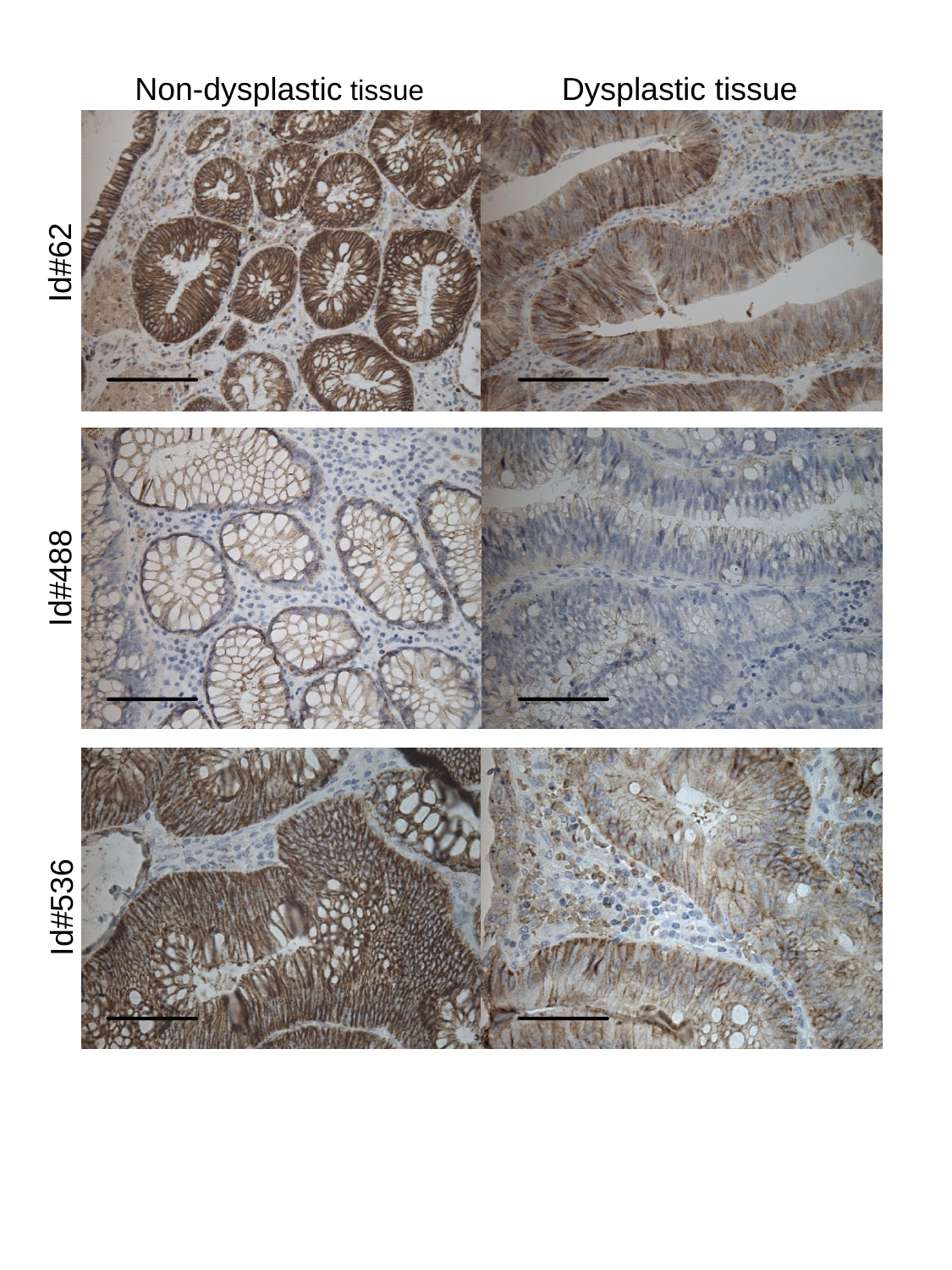

Non-dysplastic tissue
Dysplastic tissue
Id#62
Id#488
Id#536

Supplement: Additional file 1 — Immunohistochemical staining for claudin-7 in dysplastic tissue. The figure shows sections from three individuals with dysplasias. Each individual is represented in the rows marked with the numbers id#62, id#488 and id#536. The left column shows an area in the biopsy with mucosa of normal histological appearance, whereas the right column shows an area of dysplasia. Scalebars: 100 μm. [file 1471-2407-11-65-S1.PPT]

## Slide 1
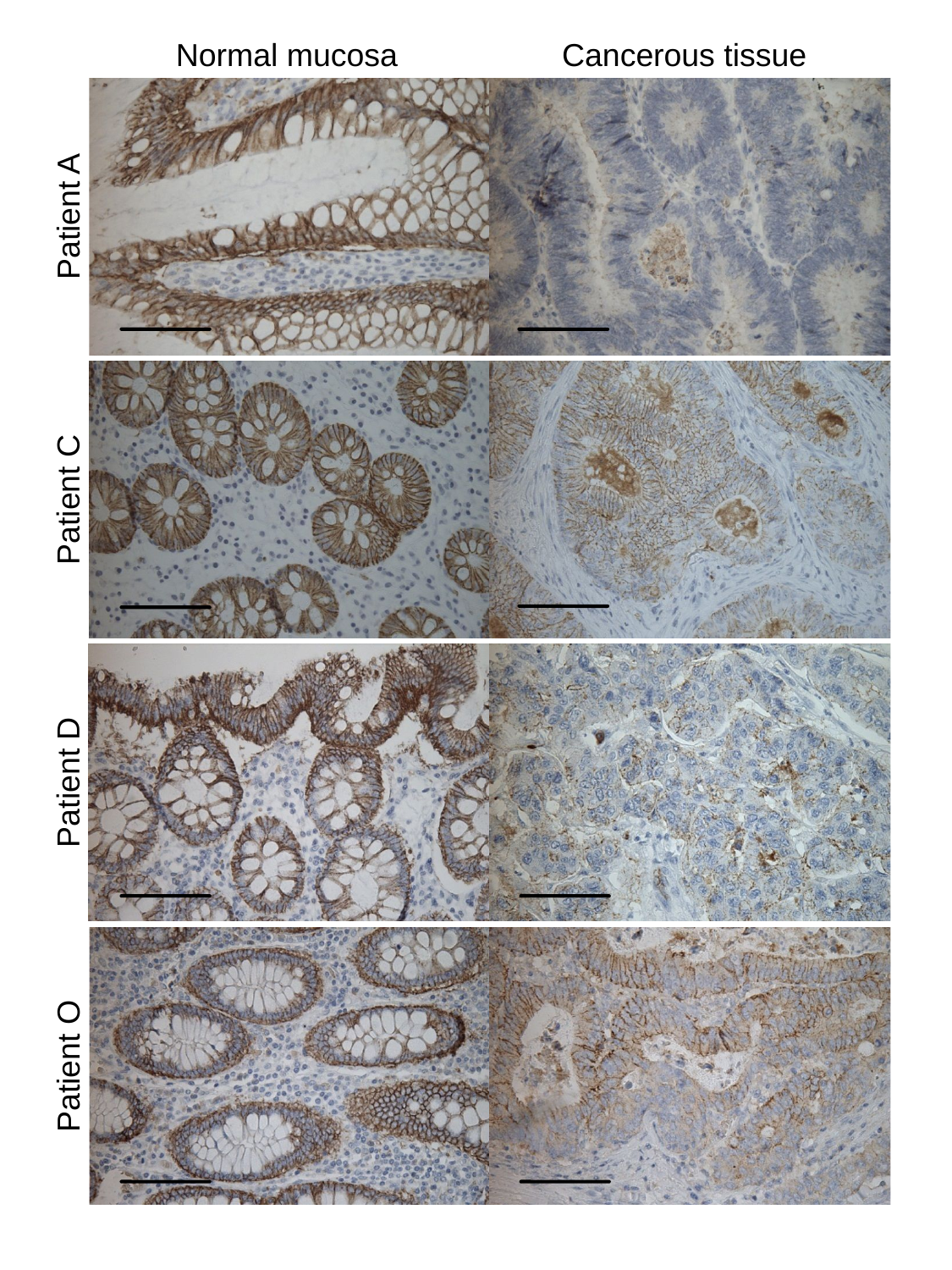

Normal mucosa
Cancerous tissue
Patient A
Patient C
Patient D
Patient O

Supplement: Additional file 2 — Immunohistochemical staining for claudin-7 in colorectal cancer. The figure represents colorectal cancer sections from four patients. Each individual is represented in the rows marked with the numbers patient A, patient C, patient D and patient O. The left column shows an area in the biopsy with mucosa of normal histological appearance, whereas the right column shows an area of cancerous tissue. Scalebars: 100 μm [file 1471-2407-11-65-S2.PPT]
